# Supplementary figures and images for: Dynamic Modulation of SO2 Atmosphere for Enhanced Fresh-Keeping of Grapes Using a Novel Starch-Based Biodegradable Foam Packaging
Source: Foods. 2023 May 31;12(11):2222. doi: 10.3390/foods12112222 (PMC10253046; doi:10.3390/foods12112222)

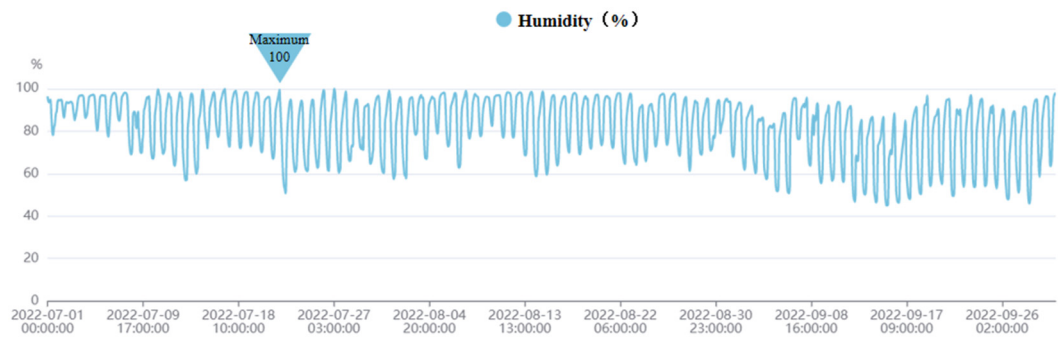

Figure S1. The humidity record from July to September in Guangzhou, China (NASA, 2022)

Supplement: Supplementary file 1 [file foods-12-02222-s001.zip › foods-2393432-supplementary.pdf]
